# Adaptation of an HIV Prevention Ambassador Program Developed in Africa for Use With Cisgender College Women in the United States: Formative Study

**DOI:** 10.2196/93268

**Published:** 2026-09-16

**Authors:** J Richelle Joe, Andrea Dunn, Carla Castro

**Affiliations:** 1 Department of Counselor Education and School Psychology, University of Central Florida, 12495 University Blvd, Orlando, FL, 32816, United States, 1 407-823-2401; 2 Let's Beehive! Inc., Orlando, FL, United States

**Keywords:** cisgender college women, HIV prevention, assessment, decision, administration and adaptation, production, topical experts, integration, training, and testing, ADAPT-ITT, adaptation, pre-exposure prophylaxis, PrEP

## Abstract

**Background:**

Cisgender college women are vulnerable to HIV transmission but rarely use biomedical prevention tools such as pre-exposure prophylaxis (PrEP). In the Orlando Metropolitan Statistical Area (MSA) of Florida, HIV vulnerability is elevated given the area’s high incidence rate (sixth among US cities). Increasing HIV and PrEP knowledge among cisgender college women can curb HIV incidence. One method of providing HIV education to cisgender college women and addressing the HIV and PrEP-related stigma is by training ambassadors through programs such as Maximizing Options to Advance Informed Choice for HIV Prevention (MOSAIC), which was developed for women and girls in Africa.

**Objective:**

The research aim was to adapt the existing MOSAIC HIV prevention ambassador program to reflect the needs of cisgender college women in Orlando MSA and to determine its acceptability, appropriateness, and feasibility for this population.

**Methods:**

The assessment, decision, administration and adaptation, production, topical experts, integration, training, and testing (ADAPT-ITT) framework for adapting evidence-based interventions guided the research. Cisgender college women (n=12) participated in focus groups that included theater testing, discussion, and surveys. Program materials were revised based on feedback, then reviewed by topical experts (n=5) who assessed feasibility and appropriateness. Data analysis was iterative; qualitative data were analyzed using rapid methods, and descriptive survey statistics complemented findings to inform curriculum revisions. Ambassadors (n=7) were trained with the adapted curriculum and provided data on acceptability and appropriateness.

**Results:**

Data from cisgender college women and topical experts indicated the program’s acceptability (mean 5.0, SD 0), appropriateness (mean 4.95, SD 0.06), and feasibility (mean 4.95, SD 0.10) and informed program adaptations. Themes from the qualitative data indicated the value of the ambassador program as well as the following recommendations: (1) provide more guidance for activities, (2) add HIV and PrEP information specific to the target population, (3) prioritize interpersonal engagement, and (4) emphasize ambassador wellness. Ambassadors reported high levels of acceptability (mean 4.75, SD 0.66) and appropriateness (mean 4.86, SD 0.38) for the adapted program after being trained and reported increases in confidence in some areas.

**Conclusions:**

Study findings provide preliminary support for the acceptability, feasibility, and appropriateness of the adapted ambassador training program. Further testing of the program can determine its use as a scalable, peer-led strategy to address HIV vulnerability and PrEP stigma in metropolitan areas with elevated HIV incidence, such as Orlando MSA.

## Introduction

### Background

Approximately 1.2 million people in the United States are currently living with HIV, and an estimated 32,100 new HIV transmissions occur each year [1]. Disparities continue to persist, with a disproportionate disease burden among sexual minority men, within racial and ethnic minoritized communities, and in the southern region of the country [2]. Additionally, both transgender and cisgender women continue to be affected by HIV, especially when they also identify as Black/African American or Hispanic/Latina [3-5]. Both the National HIV/AIDS Strategy [6] and the Ending the HIV Epidemic in the US [7] initiative have acknowledged HIV disparities and set goals to reduce new HIV diagnoses among and in priority jurisdictions through a multipronged approach that includes prevention services. Specifically, pre-exposure prophylaxis (PrEP) as a highly effective biomedical intervention for HIV prevention [8] is critical to ending HIV by 2030, and efforts to link affected groups to care along the PrEP continuum have received priority in both clinical practice and research [9-12]. However, among the populations most often newly diagnosed with HIV in the United States, PrEP services for cisgender women have been neglected. Hence, the goal of the present study was to adapt a PrEP ambassador program for cisgender college-age women in the Orlando Metropolitan Statistical Area (MSA) of Florida in an effort to establish an intervention for this underserved group.

### HIV Among Cisgender Women in Orlando MSA

Although new HIV diagnoses among women declined in the United States between 2010 and 2018, surveillance data indicate that women still account for approximately 1 in 4 new diagnoses each year [3-5]. Among those diagnosed, most are between the ages of 25 and 34 years, identify as Black, and contract HIV via heterosexual contact [3-5]. Orlando MSA ranks sixth among metropolitan areas in the country for new diagnoses, and Black women in the area have the third highest HIV prevalence rate when data are disaggregated by both race/ethnicity and gender [13,14]. Despite these data, few local HIV-serving organizations intentionally include women’s sexual health needs in their services, leaving a gap in the care continuum for this vulnerable population. Let’s Beehive! Inc, an Orlando-based nonprofit organization and community partner for the present study, *does* prioritize the HIV prevention needs of women through sexual health education, one-on-one sexual health consultations, and support groups [15]. Additionally, for the past 3 years, Let’s Beehive! Inc has collaborated on grant-funded interdisciplinary research focused on the sexual health, HIV prevention, and health care needs of Black cisgender women in Orange County, Florida [16]. Findings from this research include key themes critical to Black women’s sexual health (eg, cultural influences, experiences of gendered racism in health care, and prevailing narratives about gender roles and sexuality), as well as a concept map that includes nine domains that community stakeholders determined were important to improving Black women’s sexual health outcomes (eg, holistic healing, community accountability, and inclusive health care workforce) [17,18]. Health Department surveillance data [13,14], PrEP uptake data [19,20], and the findings of this prior research together indicate a need for additional service and research with this population in this locality, particularly in light of recent reports of twice-yearly injectable PrEP’s 100% efficacy within cisgender women [8].

### PrEP Uptake

Use of PrEP has increased exponentially from a rate of 4 users per 100,000 persons in 2012 to 180 users per 100,000 persons in 2023 [21]. In Florida broadly and Orange County specifically, PrEP usage rates are higher than the national rate at 316 and 556 per 100,000 persons, respectively [19,20]. Overall, these numbers are promising; however, when considering the sex assigned at birth and age of individuals using PrEP, significant gaps emerge. In Orange County, Florida (where Orlando MSA is located), only 11.1% of PrEP users are female and only 9.3% are aged 13 to 24 years (the lowest rate of all age categories) [20].

Research on college students indicates just how concerning low PrEP uptake is within this demographic, especially among cisgender college women. A recent analysis of data collected from over 130,000 college students in the United States indicated that only 2% of college students were on PrEP, with about 1.5% of cisgender college women taking PrEP [22]. Hence, cisgender college women account for a small percentage of all individuals making use of the most effective biomedical tool for HIV prevention. Other studies with college students have indicated that even when HIV knowledge is high, perceived vulnerability to HIV and knowledge of PrEP are often low, indicating areas for intervention [23,24]. This is particularly important for groups that have not historically been the primary target audience for HIV prevention and PrEP messages (ie, cisgender women) [23,25]. Black cisgender college women have described PrEP marketing campaigns as showing “more favoritism to gay men,” which likely contributes to the lowered perceived relevance of PrEP to their lives [25]. Low PrEP uptake among cisgender college women highlights the need to actively and intentionally engage this population at the awareness end of the PrEP care continuum, with a focus on HIV and PrEP knowledge as an avenue to facilitate PrEP linkage and stimulate movement along the continuum [26,27].

### PrEP Ambassadors for College Women

Implementation of PrEP as an evidence-based intervention for HIV prevention has included the use of PrEP navigators and HIV prevention ambassadors to increase knowledge and awareness prior to or along with the PrEP rollout [10,11]. Navigators and ambassadors serve as PrEP-knowledgeable peers who provide education, practical information, and support to individuals who are totally unaware of, interested in, or currently taking PrEP. Although navigators and ambassadors in the United States have been used efficaciously to engage sexual minority men and transgender persons in PrEP services, cisgender women have been neglected [10,11].

In the global context, peer-focused PrEP education programs have contributed to increased motivation to take PrEP, increased PrEP uptake, and improved PrEP adherence among women [9,28]. Specifically, an established HIV prevention ambassador program designed for adolescent girls and young women was updated by the Maximizing Options to Advance Informed Choice for HIV Prevention (MOSAIC) project and field tested in Zimbabwe for effectiveness in increasing the uptake of oral PrEP among adolescent girls and young women [9,29,30]. First developed in 2019 [31], the program centers the specific experiences of girls and women in its training materials by including biological aspects of HIV transmission, the multiple modalities of PrEP available to people assigned female at birth, and contextual factors that affect HIV transmission for girls and women, including lack of education and intimate partner violence. Sexual and reproductive health and rights frame the curriculum, reflecting concerns of the initial target population and expanding the relevance of the program to people living with HIV. Foundational to the program is the meaningful involvement of youth as ambassadors to their peers, with the desired outcomes being increased knowledge about HIV and PrEP among adolescent girls and young women throughout Africa [32]. Adolescent girls and young women provided input on the original curriculum as well as to subsequent updates. Initial field testing of the ambassador program yielded a 35% increase in uptake of PrEP among the women and girls who encountered ambassadors [31]. Subsequent findings indicated PrEP uptake increases of 59% and 166% as ambassadors were strategically engaged in regional and national PrEP roll-out efforts [9].

The intentionality of the MOSAIC program to address the HIV prevention needs of girls and women parallels that of the programs in the United States for sexual minority men and transgender persons and indicates a gap in programming relevant to cisgender women, including those of college age. Hence, a PrEP ambassador program for cisgender college women in Orlando MSA is needed to engage this population along the PrEP care continuum by raising awareness of PrEP and addressing PrEP-related stigma. To achieve this goal, the MOSAIC training materials would need modifications to reflect the medical, social, economic, and political landscape of the United States. Therefore, in the present study, we used the assessment, decision, administration and adaptation, production, topical experts, integration, training, and testing (ADAPT-ITT) model [33] to examine and modify the MOSAIC ambassador program and pilot test it to determine its acceptability, feasibility, and appropriateness for cisgender college women in the Orlando, Florida context.

## Methods

### Study Design

The present study sought to answer the following research question: How can an HIV prevention ambassador program designed for girls and young women in an international context be culturally and contextually adapted for cisgender college women in Orlando MSA? To answer this question, we applied the ADAPT-ITT model to the MOSAIC HIV prevention ambassador program designed for women and girls in Africa. ADAPT-ITT is a systematic and sequential process for examining and adapting evidence-based interventions (EBIs) aimed at addressing the HIV prevention and care continuums [33]. The underlying assumption of ADAPT-ITT is that EBIs require cultural and contextual relevance to maintain effectiveness, thereby requiring targeted modifications that align with the needs of new target populations and new contexts [33].

### Recruitment and Sample

Three samples of participants were recruited for this study: cisgender college women who participated in focus groups to inform adaptations of the program, topical experts who completed evaluations of the adapted program, and ambassadors who were trained with the adapted materials. Cisgender college women were recruited through criterion and snowball sampling using the following inclusion criteria: (1) at least 18 years of age, (2) cisgender woman (ie, assigned female at birth and identifies as a woman), (3) enrolled part-time or full-time in a 2-year or 4-year college in Orlando MSA. Recruitment occurred through established partnerships with campus and community organizations in Orlando MSA and included the use of flyers, direct email contact, meeting attendance, and targeted social media engagement. These efforts yielded 12 cisgender college women, each of whom participated in one focus group. Saturation was reached from the responses of these participants, which allowed us to move forward with the incorporation of their responses and the adaptation of the program. The median age for these participants was 20.5 (IQR 18-24) years. Six (50%) participants identified as Black or African American, followed by 3 (25%) who identified as White, 1 (8%) who identified as Asian, and 2 (17%) who identified as multiracial. Additionally, 3 participants indicated that they were Hispanic/Latina and 4 others indicated that they were Caribbean.

Once the program was adapted, 5 experts in sexual health, HIV prevention, and PrEP service delivery were recruited to evaluate the adapted draft of the PrEP ambassador program. These experts were identified through existing partnerships and reflected their current roles within campus and community HIV service organizations in Orlando MSA. Topical experts were recruited through criterion and snowball sampling using the following inclusion criteria: (1) at least 18 years of age and (2) paid employment or unpaid service in the areas of sexual health, HIV prevention, or PrEP delivery. Participants included an HIV tester through a college wellness center, a sexual health researcher, a community sexual health educator, a pharmacist at a clinic that prescribes PrEP, and a physician.

Once the final draft of the adapted program was complete, ambassadors were recruited for the pilot test. Inclusion criteria for ambassadors were the same as those for cisgender college women described above. Recruitment began through re-engagement with the cisgender college women who participated in the adaptation process and continued through the use of flyers and direct email contact. Recruitment yielded 7 cisgender college women who were trained as ambassadors. The median age of ambassadors was 23 (IQR 18-27) years. Six of the 7 ambassadors identified as Black or African American, and 1 identified as White. No ambassadors identified as Hispanic/Latine; however, 3 women reported being Caribbean, describing themselves as Guyanese, Haitian, and Haitian/Dominican. Ambassadors listed their programs of study as health administration, journalism, counseling, business, and pharmacy.

### Procedures

ADAPT-ITT includes 8 phases: Assessment, Decision, Administration and Adaptation, Production, Topical Experts, Training, and Testing [33]. We applied phases 3 through 8 of the 8-phase model in this adaptation (Table 1). The Assessment and Decision phases occurred prior to the initiation of the present study through the collaborative, interdisciplinary research discussed above [18]. Based on that prior research, local HIV surveillance data, and the community partner’s experiences with community engagement, cisgender college women were identified as the new target population with the MOSAIC HIV prevention ambassador program as the intervention to be adapted for the new target population in the local context. Activities during the Administration and Adaptation phase determined what aspects of the MOSAIC program would be adapted and how. During focus groups, we demonstrated elements of the program to the target population and facilitated discussions of the program’s relevance, content, and activities. Details can be found in the “Data Collection” and “Data Analysis” sections.

**Table 1.** Application of the ADAPT-ITT model to the Maximizing Options to Advance Informed Choice for HIV Prevention (MOSAIC) ambassador program.

Phase	Activities
Assessment	None: target population determined through prior research and community engagement
Decision	None: intervention selected by community partner who made the decision to adapt the program in consultation with the research partner.
Administration and adaptation	Theater testing and focus groups Quantitative and qualitative surveys to determine adaptation needs Analysis of data for adaptation plan
Production	Development of an adaptation plan that preserved core elements Draft of adapted intervention
Topical experts	Experts in sexual health, HIV prevention, and PrEP^a^ service delivery completed evaluations of adapted program
Integration	Content from topical experts integrated into final draft of the adapted intervention
Training	Final draft reviewed by research team and community partner prepared to deliver training program in pilot test
Testing	College women were trained as ambassadors using adapted curriculum and provided data on confidence, acceptability, and appropriateness

a PrEP: pre-exposure prophylaxis.

The Production phase followed the analysis of the data collected from cisgender college women in the Administration and Adaptation phase. Using that data, we developed an adaptation plan that identified the new target population (ie, cisgender college women) and the new context (ie, Orlando MSA) and described the core elements that would be preserved and the key characteristics of the program that would be adapted. The plan also identified new materials and activities to be integrated into the program and the broad aims of the adaptations to be made. Topical Experts were then engaged to capitalize on their expertise and elicit their evaluation of the program materials. During Integration, the topical experts’ input was reviewed by the research team (which included the community partner) and incorporated into a final draft of the program. The research team reviewed the final draft and the community partner prepared to deliver the training materials in the pilot test (Training). Finally, we pilot tested the adapted curriculum by using it to train PrEP ambassadors who subsequently delivered PrEP education events to their peers.

### Data Collection

Data were collected from participants during the Administration and Adaptation and Topical Expert*s* phases of the ADAPT-ITT process. Both qualitative and quantitative data from cisgender college women, topical experts, and trained PrEP ambassadors were collected and managed using REDCap electronic data capture tools (Vanderbilt University) hosted at the University of Central Florida [34,35]. REDCap is a secure, web-based software platform designed to support data capture for research studies, providing (1) an intuitive interface for validated data capture, (2) audit trails for tracking data manipulation and export procedures, (3) automated export procedures for seamless data downloads to common statistical packages, and (4) procedures for data integration and interoperability with external sources. Quantitative data included demographic data and Likert-type survey responses. Qualitative data included written responses to open-ended questions and transcripts of focus group discussion recordings with cisgender college women.

### Cisgender College Women

Participants attended a focus group which took place in person and lasted 2 hours. During the focus group, the research team provided an overview of the study, including the need to address HIV prevention in Orlando MSA, the components of the MOSAIC program, and the need to adapt the curriculum for a new population. Participants reviewed program materials (eg, infographics on HIV and PrEP) and completed sample activities from the curriculum (eg, circles of influence, character profiles, and roleplay). Then participants completed a survey to provide feedback about the program, materials, and activities. The intervention appropriateness measure (IAM) was used to assess perceived fit or relevance of the program for the new target population, and acceptability of intervention measure (AIM) was used to assess the appeal of the program to college women [36]. Additionally, participants reported the extent of their agreement with statements that the PrEP ambassador program was informative, engaging, aligned with their culture, and aligned with the culture of college women in Orlando (Likert-type scale: 1=“Completely disagree” to 5=“Completely agree”). Participants also responded to open-ended questions about what worked well, what did not work well, what could be improved, and what should be removed. After each participant completed the survey individually, they participated in a group discussion where they provided their overall impression of the PrEP ambassador program and its contents and offered suggestions that would make the program more relevant to cisgender college women in Orlando MSA.

### Topical Experts

Following analysis of data collected from cisgender college women, the curriculum was adapted, and the adapted curriculum was shared with each topical expert individually. They were asked to review the materials and complete a brief survey within 1 week of receiving the materials. The survey consisted of the IAM and the feasibility of intervention measure [36] followed by open-ended questions about the appropriateness, feasibility, and content of the adapted intervention. Open-ended questions were as follows:

- What aspects of the PrEP ambassador program do you think will work well with cisgender college women in Orlando?
- What aspects of the program do you think might be challenging with cisgender college women?
- What do you think of the cultural appropriateness of the content and activities of the program?
- Please provide any additional thoughts about the appropriateness, feasibility, and content of the PrEP ambassador program.

### PrEP Ambassadors

PrEP ambassadors were trained using the adapted curriculum over an 8-week period. They attended five 2-hour group training sessions delivered in-person by the community partner. An overview of the adapted program can be found in Multimedia Appendix 1. At the conclusion of training, participants completed a survey that included the AIM and IAM. They also responded to three items regarding their confidence: (1) helping your peers choose a PrEP method that works for them, (2) creating an action plan to increase awareness about PrEP in your community, and (3) managing stress related to your work as an ambassador. Response choices were *confidence did not increase at all*, *confidence increased some*, and *confidence increased a lot*

### Data Analysis

Analysis of the quantitative data (Likert-type survey responses) included descriptive statistics to determine the frequency, distribution, and central tendency of the data. There were no missing data. All 3 authors comprised the research team and collaboratively analyzed the qualitative data (open-ended survey responses, verbatim transcripts of focus group recordings, and focus group notes) using rapid qualitative analysis (RQA) [37]. RQA was suitable for this aspect of the study because it is an action-oriented method that allows for explanation, interpretation, and description of contextual and environmental factors that are relevant to the implementation of an intervention [38]. RQA is a team-based approach, which allowed us to capitalize on the research-practice partnership central to the study. Through this approach we engaged in data reduction that allowed us to efficiently and systematically identify relevant themes. We followed the established steps of RQA [37]: (1) develop a summary sheet template and matrix with corresponding guidelines for the completion of both, (2) generate summaries of focus group transcripts and written responses from individual participants, (3) organize and categorize data from the summary sheets into a matrix, and (4) generate themes using the matrix. The first author (principal investigator for the study) developed the summary sheet template and matrix and trained the second (community practice partner) and third (graduate student research assistant) authors on the data analysis processes. Domains on the summary sheet corresponded with focus group and survey questions (eg, impressions, content/materials, improve, remove, and add). Data were equally distributed among all 3 authors who completed the summaries independently. Direct quotations that communicated key information were included on the summaries. The first author audited the summaries for accuracy, and then the team transferred the data to the matrix to allow the data to be further analyzed both vertically and horizontally. Through this process each member of the research team identified potential themes, which were then discussed, consolidated, and agreed upon by all team members. The emergent themes and the quantitative data informed the development of the initial and final drafts of the adapted ambassador training program.

### Ethical Considerations

Prior to recruitment and data collection, all study materials and procedures were reviewed and approved by the Institutional Review Board at the University of Central Florida (STUDY00007300). All participants viewed the Explanation of Research and provided informed consent electronically prior to providing any data for the study. Additionally, participants who participated in the focus groups for the adaptation and participants who were trained as PrEP ambassadors provided verbal informed consent during the first in-person meeting with the research team. Participant identification numbers were assigned to each participant to mask their identities. Additionally, all data were stored in secure files that were only accessible to the research team. Participants received compensation for their participation as follows: cisgender college women received US $50 gift cards, topical experts received US $50 gift cards, and PrEP ambassadors received a US $1000 stipend upon completion of training and delivery of PrEP information to their peers.

## Results

Data collected from cisgender college women indicated strong support for a PrEP ambassador program for cisgender college women. Based on their responses, adaptations were made to the program content, activities, and delivery. Following the adaptation, topical experts endorsed the revised curriculum and provided insight based on their expertise.

### Cisgender College Women’s Perceptions of the PrEP Ambassador Program

Participants agreed that the program’s content and activities were informative, engaging, and aligned with the culture of cisgender college women in Orlando MSA. Responses on the IAM and AIM demonstrated that participants found the program both appropriate and acceptable. Detailed survey responses can be found in Table 2.

**Table 2.** Appropriateness, acceptability, and feasibility of pre-exposure prophylaxis (PrEP) ambassador program^a^.

	College women (n=12), mean (SD)	Topical experts (n=5), mean (SD)	PrEP ambassadors (n=7), mean (SD)
The content and activities of the PrEP ambassador program were…			
Informative	4.92 (0.29)	—^b^	—
Engaging	4.83 (0.39)	—	—
Aligned with my culture	4.67 (0.78)	—	—
Aligned with the culture of college women in Orlando	4.83 (0.58)	—	—
IAM^c^			
The PrEP ambassador program seems fitting for college women in Orlando	4.92 (0.29)	5 (0.00)	4.86 (0.38)
The PrEP ambassador program seems suitable for college women in Orlando	4.83 (0.39)	5 (0.00)	4.86 (0.38)
The PrEP ambassador program seems applicable for college women in Orlando	4.92 (0.29)	5 (0.00)	4.86 (0.38)
The PrEP ambassador program seems like a good match for college women in Orlando	4.92 (0.29)	5 (0.00)	4.86 (0.38)
AIM^d^			
The PrEP ambassador program meets my approval	5 (0.00)	—	4.71 (0.76)
The PrEP ambassador program is appealing to me	5 (0.00)	—	4.71 (0.76)
I like the PrEP ambassador program	5 (0.00)	—	4.86 (0.38)
I welcome the PrEP ambassador program	5 (0.00)	—	4.71 (0.76)
FIM^e^			
The PrEP ambassador program seems implementable.	—	5 (0.00)	—
The PrEP ambassador program seems possible.	—	5 (0.00)	—
The PrEP ambassador program seems doable	—	5 (0.00)	—
The PrEP ambassador program seems easy to use.	—	4.8 (0.45)	—

a Strong internal consistency for AIM (α=0.980) and IAM (α=0.968). Cronbach α was not computed for the FIM due to the small sample size and lack of variance in responses.

b Not applicable.

c IAM: intervention appropriateness measure.

d AIM: acceptability of intervention measure.

e FIM: feasibility of intervention measure.

Themes from the qualitative data collected from cisgender college women included (1) provide more guidance for activities, (2) add HIV and PrEP information specific to the target population, (3) prioritize interpersonal engagement, and (4) emphasize ambassador wellness. Regarding more guidance for program activities, participants suggested that more examples and demonstrations be provided for ambassadors who might not have sufficient prior knowledge to complete activities such as character profiles or role play. Additionally, they recommended that concepts like circle of influence be operationalized with examples. The recommendation to add HIV and PrEP-specific information relevant to the target population referred to surveillance data and PrEP details. For example, they were interested in infographics and other visuals that show HIV incidence in Orlando and for specific populations (eg, women, youth and young adults, and people of color). For program materials that discussed PrEP details, the participants suggested that options not approved for use with cisgender women in the United States (eg, vaginal ring and Descovy) be removed to avoid confusion. They were also interested in the PrEP side effects, possible interactions with birth control, provider information, and cost.

In terms of prioritizing interpersonal engagement, participants remarked that they enjoyed the interactive, discussion aspects of the program, especially since COVID-19 and social media have altered relational patterns. They recommended modeling the relational component necessary to being an ambassador throughout the training. Participants were adamant that ambassador wellness be emphasized. They connected wellness to clear expectations for ambassadors to reduce anxiety, as well as to preparation for confronting HIV and PrEP-related stigma. They confirmed that discussions of self-care would be relevant since ambassadors might engage in discussions of trauma, violence, and sexual assault as they engage with their peers.

### Program Adaptation

The aim of the adaptation was to develop an ambassador program that would be culturally and contextually relevant to cisgender college women in Orlando MSA, acknowledging the unique characteristics of the new target population and new context (in contrast to that of the MOSAIC program). The new target population is primarily composed of college students whose lives and schooling were interrupted and shaped by COVID-19. These women want to learn and want in-person engagement; however, they do not necessarily want homework. Additionally, this is a population that has access to information through multiple channels, including on smartphones connected to high-speed internet. Hence, they have access to information but may not know which sources are credible. Culturally and geographically, Orlando MSA is ethnically diverse and home to multiple colleges and universities. Orlando is home to one of the largest public universities in the country (enrolling over 60,000 students) as well as multiple state colleges and private institutions. Additionally, Orlando has significant Hispanic/Latine and Caribbean communities with roots in Puerto Rico, the Dominican Republic, Haiti, Jamaica, Trinidad and Tobago, Brazil, and Venezuela among other countries. Culturally held beliefs about sex, sexuality, and HIV may shape prior knowledge held by PrEP ambassadors and may contribute to biases and stigma. We considered these factors throughout the adaptation process.

Among the core elements of the MOSAIC program that we preserved were the peer-to-peer focus and the interactive components of the training. Given the isolation experienced by Generation Z as they completed high school and matriculated to college, the interpersonal engagement was considered critical, and in-person delivery of the training was prioritized. HIV basics (eg, terminology, transmission, and prevention), PrEP options, and decision-making support tools were maintained as core elements of the program.

Key characteristics of the program that we adapted related to the length of training, the content of each session, and the addition of information relevant to the geographic context (Table 3). Whereas the MOSAIC program consisted of 18 to 22 hours of seat time, we reduced the length of the training to five two-hour sessions (10 h total) plus 1 hour-long individual consultation meeting to consider the availability of the target population and their tolerance for engagement. In terms of content, we removed the sessions on human rights, getting to know our bodies, gender inequality, and violence and instead incorporated those topics into character profiles and group discussions. Additionally, handouts on PrEP options were edited to include all HIV prevention options approved by the US Food and Drug Administration (FDA) for cisgender women and removing references to the vaginal ring, Descovy, and PrEP on demand. We also added information about side effects and addressed concerns about interactions with birth control. New content incorporated into the curriculum included HIV surveillance data for Florida and Orlando MSA, HIV testing locations, local PrEP providers, and information about cost and insurance coverage of PrEP. Finally, to facilitate the development of communication skills among PrEP ambassadors, we incorporated health communication training to build confidence and reduce linguistic bias when discussing sex, HIV, and PrEP.

**Table 3.** Adaptations and program comparison.

Program element	MOSAIC^a^ program	Adaptations made	PrEP^b^ ambassador program
Target audience	Adolescent girls and young women	Narrowed target audience to respond to high needs and gaps in service	Cisgender college women
Core component	Peer training	None	Peer training
Content	Human rights Anatomy HIV/AIDS basics Gender inequality and violence Oral PrEP Bimonthly injection PrEP PrEP ring Boundary setting and self-care Responding to disclosures of violence Peer support Healthy relationships	Streamlined content to reflect prior knowledge of new target population Infused content about human rights, gender inequality, and violence into group discussions and activities such as character profiles Removed PrEP ring and added twice-yearly option	HIV/AIDS basics Oral PrEP Bimonthly and twice-yearly injection PrEP PrEP side effects, cost, and interaction with birth control Local HIV testing and PrEP resources Boundary setting and self-care Peer support Health communication skills
Seat time	18‐22 hours 21 sessions each ranging from 30 to 120 minutes	Reduced seat time and added consultation session	11 hours 5 sessions lasting 2 hours each 1-hour consultation session
Activities	Relationship and community-building Character profiles PrEP journey map Circles of influence Action and advocacy planning Self-care planning	Activities were retained and adjustments were made to how the activities were facilitated.	Relationship and community-building Character profiles PrEP journey map Circles of influence Action and advocacy planning Self-care planning

a MOSAIC: maximizing options to advance informed choice.

b PrEP: pre-exposure prophylaxis.

### Topical Experts’ Perceptions of the PrEP Ambassador Program

Following their review of the adapted curriculum, topical experts endorsed the appropriateness and feasibility of the PrEP ambassador program (Table 2). Their qualitative responses provided additional support for the program as well as suggestions for improving its relevance for the new target population. Regarding aspects of the program that they thought would work well, topical experts discussed the surveillance data and PrEP details that would provide evidence-based medical knowledge to support cisgender college women’s decision-making. One topical expert commented on the delivery of the content noting, “I think the program being broken into multiple small sessions with very digestible information will work well for engagement and retention.” This response aligns with our attempts to consider the tolerance cisgender college women would have for this program in terms of time commitment and depth of information. Another topical expert commented on the importance of the program for cisgender college women given that PrEP has been largely marketed to men, stating that, “assuring to their peers that PrEP is not only for homosexual men or MSM” was an asset of the program.

In response to questions about aspects of the program that might be challenging and the cultural appropriateness of the program, topical experts noted the amount of information to be delivered, stigma associated with both sex and HIV, and the inclusive nature of the program. Topical experts cautioned about overloading PrEP ambassadors with scientific information about HIV, AIDS, and PrEP, “I think some of the aspects in Session 2 (PrEP Methods) may be a challenge due to there being an extensive amount of information given but good information” and “I think getting into the specifics of PrEP such as which medications and some of the pathophysiology of HIV vs AIDS may be slightly difficult for college women to grasp. Usually, we learn about these details in medical school.” Both comments are relevant given the potential for PrEP ambassadors to have varying levels of prior knowledge about HIV and PrEP, as well as different levels of interest in the details of each medication. Topical experts reported that they found the program to be culturally appropriate for cisgender college women in Orlando MSA. One expert mentioned the visual appropriateness of the materials stating, “I think the information provided in this program is culturally appropriate and inclusive. The information is relevant for women of all ages across all backgrounds. The images and graphics are inclusive.” Another expert specifically referenced the Haitian community stating, “I think it is culturally appropriate, especially in the Haitian population where medicine can sometimes be paternalistic and now through transparency and education, some of the myths can be dispelled.” This insight is key given the sizable community of Haitians in Orlando MSA.

### PrEP Ambassador’s Perceptions of the PrEP Ambassador Program

After completing the training with the adapted program curriculum, PrEP ambassadors rated the program highly for acceptability and appropriateness (Table 2). Additionally, most ambassadors reported that their confidence increased due to the training. Specifically, 86% (n=6) of ambassadors responded that their confidence increased a lot or some to the items “helping your peers choose a PrEP method that works for them” and “creating an action plan to increase awareness about PrEP in your community.” On the item “managing stress related to your work as an ambassador,” 57% (n=4) reported their confidence increased a lot, 29% (n=2) reported that it increased some, and 14% (n=1) reported that their confidence did not increase at all. Qualitative responses from the ambassadors indicated their goals following ambassador training. Responses included their commitment to sharing information about how HIV is transmitted and how it can be prevented with PrEP, “It is important for people to understand what HIV is and that it is a disease that can replicate fast without medication. I want everyone to know that taking PrEP decreases your chances of getting HIV” and “I plan on spreading the message of everything I learned about PrEP to people I know, so they’re not oblivious to HIV and how it’s transmitted through sex!” One ambassador focused on her willingness to broach the conversation with others, saying, “I plan to be more open about the topic of HIV and PrEP.” Another ambassador reported that she plans to apply the information in her future career as a counselor, “I aspire to implement the information I learned into the counseling field and apply the information I learned to my future clients if the situation is applicable.” An ambassador even focused on the application of the information in her own life stating, “I plan to be cautious when I am in a relationship.”

## Discussion

### Principal Findings

The purpose of this study was to adapt an existing HIV prevention ambassador program to be used with cisgender college women in Orlando MSA and to assess its acceptability, appropriateness, and feasibility. The adapted curriculum retained core elements of the MOSAIC program, such as core content (eg, HIV basics, PrEP options, and decision-making), the peer-to-peer focus, and interactive components of the training. Key characteristics were adapted for cultural and contextual alignment with the needs and expectations of cisgender college women in Orlando MSA, and health communication training was added to strengthen ambassadors’ skills. Both the cisgender college women and topical experts who reviewed and provided input on the curriculum responded favorably to the program itself as well as to the content and materials within it as evidenced by high scores for acceptability, appropriateness, and feasibility. Following the pilot test, trained ambassadors also endorsed the acceptability and appropriateness of the program and noted increases in their confidence with respect to key training outcomes (ie, helping peers choose a PrEP method, creating a plan to share PrEP information, and self-care). These findings indicate that approaches to facilitate engagement with PrEP services that have been used with cisgender men and transgender women in the United States and cisgender women in Africa have applicability for cisgender women in the United States.

### Comparison With Prior Work

Additionally, the findings indicate cultural and contextual nuance necessary for using a program developed in Africa with cisgender college women in the United States. The participants in this study had questions about the cost and side effects of PrEP, as well as potential interactions with birth control. These concerns align with documented barriers to PrEP uptake among women in the United States and reflect mistrust of both the health care system and the pharmaceutical industry [39,40]. Based on participants’ responses, it was important to incorporate this information into the training materials, including handouts and worksheets that the ambassadors would use to share with their peers. Similarly, participants’ suggestion that only formulations of PrEP approved for cisgender women in the United States be included on written materials is reflective of context as well. Participants stated, and we agreed, that clarity regarding available options for the target population was critical as confusion might contribute to hesitance to engage with PrEP.

Study findings regarding the interactive aspect of the ambassador program underscored the value of incorporating health communication into the training. Participants’ comments about the effects of COVID-19 on interpersonal relationships echo extant research from the height of the COVID-19 pandemic in 2020 and the years since. Social disconnectedness, loneliness, and altered communication types resulting from shelter-in-place directives and social distancing present both challenges and opportunities for the development of communication skills and the utilization of new methods of communication [41,42]. The integration of health communication into the PrEP ambassador program addresses interpersonal challenges that might be lingering for cisgender college women whose high school and early college experiences were shaped by COVID-19.

### Limitations

Although the study findings resulted in an adapted program to prepare cisgender college women to be PrEP ambassadors, it is important to note the limitations of the research. First, by focusing this adaptation on cisgender college women, we narrowed the target population of the original MOSAIC curriculum which was intended for adolescent girls and young women irrespective of their status as students. This was an intentional choice, yet it presented challenges regarding recruitment, availability of participants, and some demographic variables, such as age. Because colleges and universities operate on their own calendars, recruitment was challenging during the summer session, thus limiting our sample size. Additionally, by using enrollment in a college or university as inclusion criterion rather than age, we encountered nontraditional college students who were older than their peers. Using an age limit might have ensured participants were the expected college age; however, this might have excluded older women who interact with traditional college–aged women in classes and other social settings. Solving this conundrum may not be possible given the age diversity that exists on college campuses; still, this is an important consideration for understanding our findings and for designing future studies that target cisgender college women. Finally, although the sample size for this study was appropriate for formative work, the near-uniform high ratings on the acceptability, feasibility, and appropriateness scales indicate the need for further study of the program to ensure representativeness of the target population. In Orlando MSA, where the Hispanic/Latine population is sizeable, this would require intentional recruitment of cisgender women who identify as Hispanic/Latine, given that none were trained as ambassadors in this study.

### Conclusions

Despite the study limitations, the findings indicate that the adapted PrEP ambassador training curriculum is appropriate and acceptable to cisgender college women in Orlando MSA, a high incidence locality. Trained PrEP ambassadors reported increased confidence in their ability to share HIV and PrEP information with their peers. Further testing of the PrEP ambassador program can refine its delivery and provide preliminary support for its use. With additional empirical support, the use of trained PrEP ambassadors to disseminate information about PrEP to cisgender women across the lifespan, regardless of college enrollment, could be an important strategy to facilitate increased uptake of PrEP by cisgender women. Whereas translational science to introduce new formulations for PrEP is valuable and FDA approval of a vaginal ring would provide more options for women, the PrEP ambassador program can strategically address the knowledge gap that exists for cisgender women in the United States who have low awareness of the PrEP options available to them.

## Supplementary material

10.2196/93268 Multimedia Appendix 1 Adapted pre-exposure prophylaxis (PrEP) ambassador training program overview**.**

## Abbreviations

ADAPT-ITT: assessment, decision, administration and adaptation, production, topical experts, integration, training, and testing
AIM: acceptability of intervention measure
EBI: evidence-based intervention
FDA: US Food and Drug Administration
IAM: intervention appropriateness measure
MOSAIC: Maximizing Options to Advance Informed Choice for HIV Prevention
MSA: Metropolitan Statistical Area
PrEP: pre-exposure prophylaxis
RQA: rapid qualitative analysis
