# Supplementary material for: Adaptation of an HIV Prevention Ambassador Program Developed in Africa for Use With Cisgender College Women in the United States: Formative Study
Source: JMIR Form Res. 2026 Sep 16;10:e93268. doi: 10.2196/93268 (PMC13581142; doi:10.2196/93268)
Supplement: Supplementary file 1 [file formative-v10-e93268-s001.docx]

**Appendix**

**Adapted PrEP Ambassador Training Program Overview**

**(Each session will last two hours)**

**Session 1: Training Introduction and Foundational Knowledge**

**Objectives: 1) To build community among future PrEP ambassadors and 2) To establish baseline knowledge about HIV/AIDS**

- Introductions and icebreakers
- Hopes and fears; Needs and responsibilities
- HIV surveillance data for U.S. and Orlando MSA
- HIV and AIDS Basics: terminology, transmission, prevention, and treatment

**Session 2: PrEP Methods**

**Objectives: 1) To explain how PrEP and PEP work and 2) To identify multiple PrEP options available to cisgender women in the U.S.**

- PrEP Methods and combination prevention
- Oral and injectable PrEP essential knowledge
- PEP and doxy PEP
- Talking about PrEP methods

**Session 3: PrEP Methods**

**Objectives: 1) To develop skills that will support cisgender women’s PrEP decision-making and 2) To identify methods to support access to PrEP and adherence to a PrEP regimen**

- Deciding to use PrEP
- Getting PrEP
- Taking and staying on PrEP

**Session 4: Ambassador Skills**

**Objectives: 1) To develop peer engagement skills, 2) To establish a self-care plan, and 3) To begin a plan for sharing HIV and PrEP information with others**

- Circles of Influence
- Boundary setting and self-care
- Peer support skills
- Planning a PrEP event

**Session 5: Communication and Closing**

**Objectives: 1) To practice health communication skills and 2) To finish plan for sharing HIV and PrEP information with others**

- Effective health communication skills
- Planning a PrEP event
- Ambassador graduation
